# Supplementary material for: Phosphorylation-induced SUMOylation promotes Ulk4 condensation at the ciliary tip to transduce Hedgehog signal
Source: J Cell Sci. 2025 May 19;138(20):jcs263695. doi: 10.1242/jcs.263695 (PMC12148026; doi:10.1242/jcs.263695)
Supplement: Supplementary information [file joces-138-263695-s1.pdf]

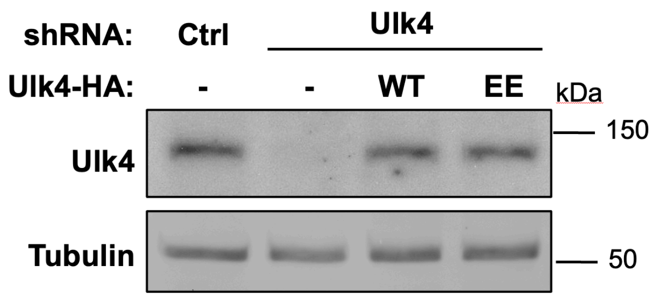

Fig. S1. Western blot analysis of Ulk4 transgenes

NIH3T3 cells with endogenous Ulk4 depleted by shRNA were infected with the indicated human Ulk4 lentiviral constructs. Cell lysates were subjected to western blot analysis with the indicated antibodies. Of note, Ulk4-WT-HA and Ulk4-EE-HA were expressed at levels comparable to the endogenous Ulk4 level.

Journal of Cell Science • Supplementary information

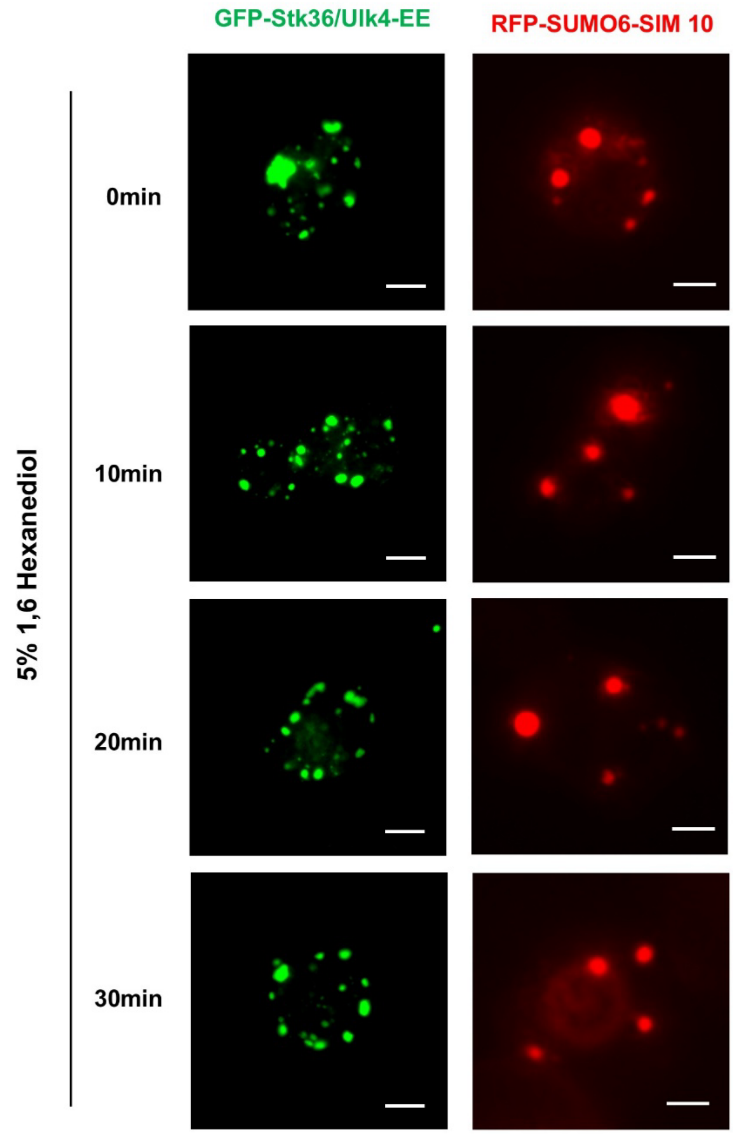

Fig. S2. Stk36/Ulk4 condensates are resistant to 1, 6-hexanediol
Representative images of HEK293T cells expressing the indicated constructs before and after treatment with 5% 1, 6-hexanediol for 10, 20, and 30 minutes. Scale bars are 5 mm.

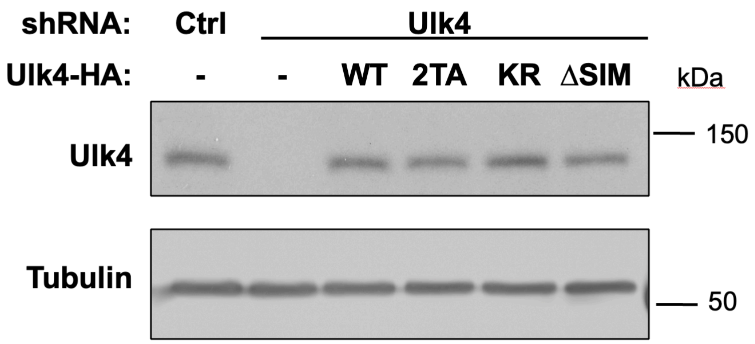

Fig. S3. Western blot analysis of Ulk4 transgenes

NIH3T3 cells with endogenous Ulk4 depleted by shRNA were infected with the indicated human Ulk4 lentiviral constructs. Cell lysates were subjected to western blot analysis with the indicated antibodies. Of note, Ulk4-WT-HA, Ulk4-2TA-HA, Ulk4-KR-HA, and Ulk4-ΔSIM-HA were expressed at levels comparable to the endogenous Ulk4 level.

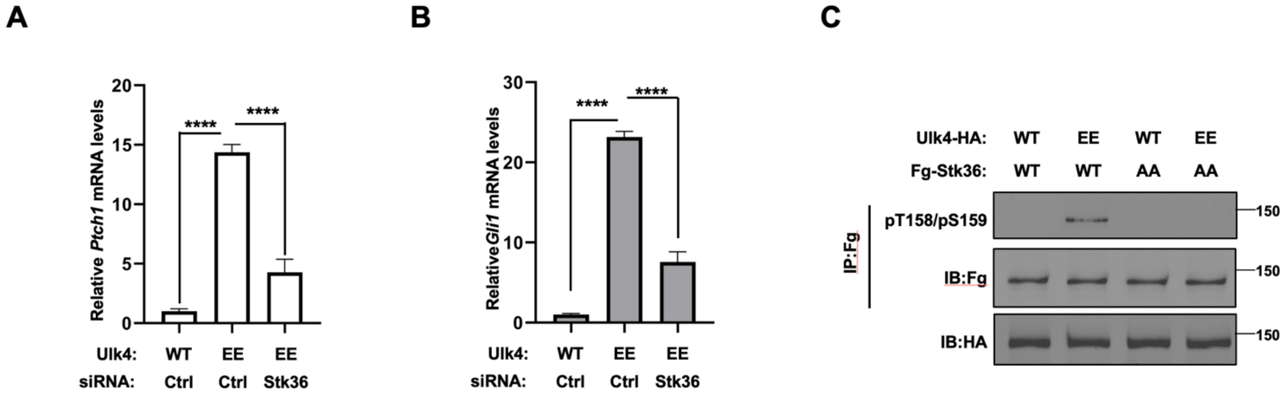

Fig. S4. The constitutive pathway activity of Ulk4-EE depends on Stk36

(A, B) Relative *Ptch1* (A) and *Gli1* (B) mRNA levels in NIH3T3 cells infected with the indicated Ulk4 lentiviral constructs and treated with control (Ctrl) or Stk36 siRNA. (C) Western blot analysis of Stk36 phosphorylation on pT158/pS159 in NIH3T3 cells co-infected with the indicated Ulk4 and Stk36 lentiviral constructs. Data are mean ± SD. ****p < 0.0001 (one-way ANOVA test). Results in (A) and (B) are representatives of three independent experiments.

Journal of Cell Science • Supplementary information
